# High incidence of septic shock caused by *Streptococcus pneumoniae* serotype 3 - a retrospective epidemiological study

**DOI:** 10.1186/1471-2334-13-492

**Published:** 2013-10-22

**Authors:** Jonas Ahl, Nils Littorin, Arne Forsgren, Inga Odenholt, Fredrik Resman, Kristian Riesbeck

**Affiliations:** 1 Department of Laboratory Medicine Malmö, Medical microbiology, SE-205 02 Malmö, Sweden; 2 Department of Clinical Sciences, Infectious Diseases Research Unit, Malmö, Lund University, SE-205 02 Malmö, Sweden

**Keywords:** Invasive pneumococcal disease, Sepsis, Serotype 3, Serotype 19F, *Streptococcus pneumoniae*

## Abstract

**Background:**

More than 90 immunologically distinct serotypes of *Streptococcus pneumoniae* exist, and it is not fully elucidated whether the serotype is a risk factor for severity of invasive pneumococcal disease (IPD). Our hypothesis is that serotypes differ in their capacity to cause septic shock.

**Methods:**

We performed a retrospective study in Southern Sweden based upon 513 patients with IPD in the pre-vaccine era 2006–2008. The serotype, co-morbidity, and sepsis severity were determined. Serotypes were compared to serotype 14 as a reference and grouped according to their invasive potential, that is, high (serogroups 1, 5 and 7), intermediate (serogroups 4, 9, 14 and 18) and, finally, low invasive potential (serogroups 3, 6, 8, 15, 19, 23 and 33).

**Results:**

Patients with *S. pneumoniae* serotype 3 had significantly more often septic shock (25%, odds ratio (OR) 6.33 [95% confidence interval (CI) 1.59-25.29]), higher mortality (30%, OR 2.86 [CI 1.02-8.00]), and more often co-morbidities (83%, OR 3.82 [CI 1.39-10.54]) when compared to serotype 14. A significant difference in age and co-morbidities (*p*≤0.001) was found when patient data were pooled according to the invasive potential of the infecting pneumococci. The median age and percentage of patients with underlying co-morbidities were 72 years and 79%, respectively, for serogroups associated with low invasiveness, 68 years and 61%, respectively, for serogroups with intermediate invasiveness, and, finally, 62 years and 48%, respectively, for serogroups with high invasiveness. No difference in sepsis severity was found between the three groups.

**Conclusions:**

*S. pneumoniae* serotype 3 more often caused septic shock compared to serotype 14. Our results support the hypothesis that serotypes with high invasiveness mainly cause IPD in younger patients with less co-morbidities. In contrast, serogroups with low and intermediate invasive potential mostly cause IPD in the elderly with defined co-morbidities, and thus can be considered as opportunistic.

## Background

*Streptococcus pneumoniae* is a common colonizer of the human respiratory tract and is associated with morbidity and mortality worldwide. The species is the leading bacterial cause of acute otitis media, sinusitis, pneumonia and a major cause of invasive infection, such as meningitis and sepsis, *i.e.*, invasive pneumococcal disease (IPD) [1].

*S. pneumoniae* is equipped with several virulence factors of which the composition of the polysaccharide capsule, *i.e.*, the serotype, is considered to be the most important one [2]. Many serogroups are divided into serotypes and to date more than 90 serotypes have been defined. Variation in nasopharyngeal carriage rate, invasive capacity and disease rate are related to specific serotypes and serogroups. The invasive disease potential of a particular serotype is related to the tendency to cause IPD while colonizing the nasopharynx [3]. A significant inverse correlation between invasive disease and carriage rate has been observed [3]. In addition, other studies have shown that *S. pneumoniae* with a low invasive potential is more likely to infect the older population and results in IPD with a more severe outcome [4,5].

An important issue is whether the serotype is a risk factor for IPD severity. Some studies suggest that host factors such as underlying disease and age are more important determinants than a particular serotype [6,7]. In contrast, certain serotypes are associated with more severe outcome and mortality even after adjustment for relevant host factors [4,8-10]. A meta-analysis by Weinberger *et al*. in 2010 supports that the serotype plays an important role in determining the outcome of bacteremic pneumonia [11]. These authors also found that particularly serotypes 3 and 19F, which have a thicker capsule *in vitro* as measured with digital fluorescence microscopy, more frequently are associated with a fatal outcome. Serotype 3 is independtly associated with a higher incidence of septic shock as revealed in a study by Garcia-Vidal *et al.*[12]. In parallel, experimental animal studies revealed that serotypes with a thicker capsule are more virulent [13].

Clinical studies on sepsis severity in patients with IPD that are correlated to the pneumococcal serotype are scarce. To reveal whether or not serotypes differ in virulence, we therefore chose septic shock as the primary endpoint. A putative relationship between serotype and the frequency of septic shock was studied in a defined population in Southern Sweden (years 2006–2008). In addition, mortality, age and co-morbidities were carefully documented. The prevalence of penicillin non-susceptible pneumococci (PNSP) is only 3.0% among nasopharyngeal carriage disease strains in the study area, and a conjugated pneumococcal vaccine (PCV) not included in the national vaccine program until 2009. These two confounding factors could thus be excluded in our analyses. Interestingly, we found that the frequency of septic shock differed between serogroups and that serotype 3 significantly more often caused septic shock as compared to serotype 14.

## Methods

### Identification of patients

All cases with IPD in Sweden are reported to the Swedish Institute for Communicable Disease Control. The county of Skåne in southern Sweden including 1.2 million inhabitants was the study area. The pneumococcal vaccine coverage was low in this community, and in 2007 1,366 doses of the 23-valent polysaccharide vaccine and 1,287 doses of the 7-valent conjugated vaccine (PCV7) were administered according to sales statistics (data on file).

In this retrospective epidemiological study, 628 patients with IPD were identified during the years 2006–2008. One hundred thirteen cases (58 men and 55 women, median age 65-years) were excluded since isolates had been lost, and two cases were not included due to absence of full medical records. The 28-day crude mortality rate for the excluded patients was 13%.

### Culture conditions and serotyping

Cultures, identification, PCR and antimicrobial susceptibility tests were performed according to national guidelines [14,15]. Serotyping was done either with a capsular reaction test or a gel diffusion method with antisera from Statens Serum Institute (SSI; Copenhagen, Denmark) [16,17]. Further serotyping was performed using the Quellung reaction with antisera (SSI) [18] for the serogroups 6, 7, 9, 18, 19, and 23 included in the conjugate vaccines. In total, 4 isolates were missing, 10 isolates belonging to a vaccine serogroup were not successfully serotyped since the isolates were dead after thawing.

### Patient and disease characteristics

The following data were collected from medical records; age, gender, infection foci, admission to Intensive Care Unit (ICU), septic shock, co-morbidities, immunosuppressive treatment, ongoing alcohol abuse or smoking, and, finally, mortality after 28 days and after one year. Co-morbidities were noted when a diagnosis was specified in the medical records and included diagnoses were divided into the categories cardiovascular, lung, neurological, hematologic and autoimmune disease, liver and renal failure, diabetes mellitus, solid cancer, splenectomy, and HIV. Diagnoses in medical records after in-patients hospital treatment in the county of Skåne have proven to represent a high validity [19].

Severe sepsis and septic shock during the hospitalization were defined according to criteria in the surviving sepsis campaign [20]. Only objective parameters were included in the analysis and absence of parameters was thoroughly recorded. The sepsis severity grading in relation to serotype was blinded for the examiner. Each case was individually reviewed and data on previous organ function was carefully searched for in the medical records. The sepsis criteria were only considered fulfilled if there was no other explanation for the change in organ function other than sepsis. Oxygen saturation obtained by pulse oximetry was converted to PaO_2_ according to a reference guide when required [21]. Septic shock was defined as severe sepsis with persisting hypotension despite adequate fluid resuscitation with at least 500 ml intravenous fluid given in 30 minutes. A patient that fulfilled the criteria for septic shock was recorded even if the SIRS criterion was not fulfilled.

### Statistical analyses

All data were analysed with SPSS, version 20. Potential association between serotype and age, mortality, ICU care, and frequency of septic shock was determined in two different ways;

1. Serotypes were divided into three different classes depending on their invasive potential in children according to a meta-analysis by Brueggeman *et al.*[3], where carriage rates of serogroups and serotypes were compared with their rates of IPD. Brueggeman and collaborators selected serotype 14 as a reference since it is a single serotype with no subtypes, is among the most prevalent invasive and carriage serotypes, and, finally, shows no evidence of heterogeneity. Moreover, Brueggeman *et al.* determined odds ratio (OR) with 95% confidence interval (CI) compared to serotype 14. Pneumococcal serogroups were divided into having a high, intermediate or low invasive potential based upon this analysis. An OR >1 was associated with serogroups included in the highly invasive serogroups [1,5,7]. Odds ratio 0.5-1 included the intermediate invasive serogroups [4,9,14,18], and OR <0.5 comprised the low invasive serogroups (3, 6, 8, 15, 19, 23 and 33). Generalized Fisher’s exact test was used to compare binary data between the three groups and Kruskal Wallis test for ordinal data.

2. Serotypes were compared one by one to serotype 14 as a reference based upon the rationale described above. Fisher’s test was included to compare binary data and Mann-Whitney’s test was used for ordinal data. Odds ratios, 95% CI and *p*-values were consequently calculated.

### Ethical approval

This study was approved by The Regional Ethical Review Board in Lund, Lund University, Sweden (2011/65). Individual informed consent was not recommended by the Ethical Board. The study was retrospective and all personal data has been handled under strict secrecy and the results in our study cannot be connected to individual patients.

## Results

### Invasive pneumococcal disease in south Sweden is dominated by serotypes 3, 4, 7F, 9V and 14

In total, 513 patients were included in the analysis which consisted of 246 men and 267 women. Median age was 67 years (range 19–101 years). The majority of patients (87%) were diagnosed with pneumonia, 5% with meningitis, 8% percent with unknown infection focus, and 3% had two infection foci. Otitis, arthritis, osteitis, endocarditis and epiglottitis were rare diagnoses.

We further analysed all pneumococcal strains, and the distribution is presented in Table 1. In our collection were 10 isolates non-typable. Serotypes 7F and 14 were the most abundant representing 12.7% and 11.7% of the isolates, respectively. The five most common serotypes consisted of 50% of all isolates and the 12 most common serotypes comprised 76% of all isolates. Antimicrobial resistance was as expected very low, and only 2.5% of patients were infected with PNSP. The MIC-values for PcG was 2 mg/L in one, 1 mg/L in four and 0.125-0.5 mg/L in 8 of the pneumococcal isolates.

**Table 1:** Characteristics of patients with IPD by infecting serotype and statistical comparison in relation to infection with serotype 14

		**Septic shock**		**28 day mortality**		**Admitted to the ICU**		**Any co-morbidity**		**Two or more co-morbidities per patient**		**Median age**	
**Serotype (no)**	**Percent of all isolates**	**No (%)**	**OR (CI)**	**No (%)**	**OR (CI)**	**No (%)**	**OR (CI)**	**No (%)**	**OR (CI)**	**No (%)**	**OR (CI)**	**years (range)**	***p-*** **value**
**14 ( *n *** **=60) Reference**	11.7	3 (5)	1	8 (13)	1	8 (13)	1	35 (58)	1	10 (17)	1	70 (20–94)	*-----*
**1 ( *n *** **=14)**	2.7	0 (0)	*-----*	0 (0)	*-----*	0 (0)	*-----*	4 (29)	0.31 (0.09-1.07)	0 (0)	*-----*	**49 (32–68)**	**<0.001**
**3 ( *n *** **=36)**	7.0	**9** (25)	**6.33 (1.59–25.29)**	**11 (30)**	**2.86 (1.02–8.00)**	8 (22)	1.86 (0.63–5.48)	**30 (83)**	**3.82 (1.39–10.54)**	**15 (62)**	**3.57 (1.38–9.22)**	75 (40–94)	0.530
**4 ( *n *** **=55)**	10.7	6 (11)	2.32 (0.55–9.79)	3 (55)	0.37 (0.09–1.49)	7 (13)	0.95 (0.32–2.81)	30 (55)	0.92 (0.44–1.92)	15 (27)	1.86 (0.76–4.62)	66 (20–91)	0.550
**6A ( *n *** **=20)**	3.9	0 (0)	*-----*	3 (15)	1.15 (0.27–4.82)	0 (0)	*-----*	14 (70)	1.78 (0.60–5.28)	**11 (55)**	**6.11 (2.01–18.58)**	73 (34–91)	0.669
**6B ( *n *** **=24)**	4.7	1 (4)	0.83 (0.08–8.36)	1 (4)	0,28 (0,03–2.39)	3 (12)	0.93 (0.22–3.84)	18 (75)	2.29 (0.80–6.59)	**9 (37)**	**3.00 (1.03–8.74)**	73 (34–97)	0.760
**7F ( *n *** **=65)**	12.7	7 (11)	2.29 (0.56–9.31)	6 (9)	0.66 (0.22–2.03)	12 (18)	1.47 (0.56–3.89)	33 (51)	0.74 (0.37–1.50)	16 (25)	1.63 (0.68–3.95)	**63 (19–95)**	**<0.001**
**8 ( *n *** **=18)**	3.5	2 (11)	2.37 (0.36–15.46)	2 (11)	0,81 (0.15–4.22)	2 (11)	0.81 (0.16–4.22)	11 (61)	1.20 (0.41–3.53)	5 (28)	1.92 (0.56–6.61)	64 (49–86)	0.051
**9V ( *n *** **=41)**	8.0	4 (10)	2.05 (0.43–9.71)	4 (10)	0.70 (0.20–2.50)	8 (20)	1.58 (0.54–4.61)	27 (66)	1.42 (0.65–3.39)	10 (24)	1.61 (0.60–4.32)	69 (41–97)	0.381
**18C ( *n *** **=16)**	2.9	2 (12)	2.71 (0.41–17.83)	4 (25)	2.17 (0,56–8.40)	4 (25)	2.17 (0,56–8.40)	**14 (88)**	**5.35 (1.12–25.66)**	**11 (69)**	**11.00 (3.13–38.64)**	70 (37–85)	0.199
**19A ( *n *** **=7)**	1.4	0 (0)	*-----*	0 (0)	*-----*	1 (14)	1.08 (0.11–10.22)	6 (86)	4.59 (0.52–40.50)	2 (28)	2.00 (0.34–11.80)	70 (34–91)	0.845
**19F ( *n *** **=16)**	4.9	3 (19)	4.38 (0.79–24.24)	4 (25)	2.17 (0.56–8.39)	**8 (50)**	**6.50 (1.90–22.25)**	13 (81)	3.31 (0.85–12.85)	7 (44)	**3.89 (1.17–12.89)**	64 (24–91)	0.395
**23F ( *n *** **=25)**	4.9	0 (0)	*-----*	0 (0)	*-----*	1 (4)	0.27 (0.03–2.29)	**21 (84)**	**4.1 (1.23–13.13)**	**12 (48)**	**4.61 (1.63–13.03)**	73 (23–101)	0.835

The 28 serotypes/serogroups with low incidence are not included in the table and were in descending order: 9N (*n*=11), 22 (*n*=11), 12 (*n*=10), 11 (*n*=9), 6C (*n*=9), 17 (*n*=4), 38 (*n*=4), 33 (*n*=4), 20 (*n*=4), 15 (*n*=3), 9A (*n*=3), 31 (*n*=3), 23A (*n*=3), 7 (*n*=3), 9 (*n*=3), 7A (*n*=2), 35 (*n*=2), 18 (*n*=2), 13 (*n*=2), 10 (*n*=2), 8F (*n*=1), 5 (*n*=1), 28 (*n*=1), 27 (*n*=1), 23B (*n*=1), 23 (*n*=1), 19 (*n*=1), and, finally, 18F (*n*=1). Ten isolates were nontypable (1.9%) and 4 isolates were missing.

β-lactam antibiotics were included in the empirical treatment of 488 patients, and 113 patients were treated with two different classes of antibacterial agents. According to the Swedish guidelines, penicillin is the recommended empirical treatment against pneumonia, and was consequently given to 218 patients. Patients not administered β-lactam antibiotics were treated with clindamycin (*n*=12), erythromycin (*n*=5), doxycycline (*n*=3), gentamicin (*n*=2), or vancomycin (*n*=2). One patient was not at all treated with antibacterial agents.

### Pneumococcal serotype 3 causes more often septic shock compared to serotype 14

The 28-day crude mortality rate for all patients was 12.3%, and after one year 19.5% of the patients were dead. One patient infected with a PNSP having MIC 0.125 mg/L for PcG did not survive. A difference, albeit not significant, was revealed between 28-day mortality rate in serotype/ serogroups when divided into high (7%), intermediate (13%) and low (14%) invasive potential (Table 2). Serotype 3 was the only serotype with a significantly higher mortality (30%) when compared to the reference serotype 14 (13%) (Table 1). Among the 12 most common serotypes, no cases of mortality were noted amongst patients infected with serotype 23F, 1 or 19A. The mortality rate after 28 days, the incidence of septic shock and furthermore ICU care for the most common co-morbidities are presented in Table 3.

**Table 2:** Characteristics of patients with IPD grouped according to invasive potential of the infecting pneumococcal serotype

**Invasive disease potentialª (no)**	**Septic shock (%)**	**28 day mortality (%)**	**Admitted to the ICU (%)**	**Any co-morbidity (%)**	**Two or more co-morbidities per patient (%)**	**Median age (years; range)**
**High**^**b **^**(*n *** **= 85)**	7 (8%)	6 (7%)	12 (14%)	41 (48%)	19 (22%)	62 (19–95)
**Intermediate**^**c **^**(*n *** **= 192)**	18 (9%)	25 (13%)	29 (15%)	117 (61%)	52 (27%)	68 (20–97)
**Low**^**d **^**(*n *** **= 169)**	17 (10%)	24 (14%)	24 (14%)	133 (79%)	74 (44%)	72 (23–101)
***p*-value**^**e**^	*p*=0.911	*p*=0.293	*p*=0.984	***p*** **<0.001**	***p*** **<0.001**	***p*** **<0.001**

ªSerogroups divided into groups depending of invasive potential according to Brueggeman *et al.*[3]. Sixty-seven IPD cases were excluded since the serotype was not included in this classification.

^b^High invasive disease potential includes serotypes in serogroups 1, 5, and 7.

^c^Intermediate invasive disease potential includes serotypes in serogroups 4, 9, 14 and 18.

^d^Low invasive disease potential includes serotypes in serogroups 3, 6, 8, 15, 19, 23, and 33.

^e^Univariate *p*-value for differences among the groups (high, intermediate and low invasive potential).

**Table 3:** Prevalence of co-morbidities, associated mortality, incidence of septic shock, and ICU admission per co-morbidity

**Type of co-morbidity**^**a**^	**Number of patients (%)**	**28 day mortality (%)**	**Septic shock (%)**	**Admitted to the ICU (%)**
Cardiovascular disease	136 (27%)	25 (18%)	18 (13%)	26 (19%)
Lung disease	125 (24%)	20 (16%)	20 (16%)	26 (21%)
Neurological disease	67 (13%)	12 (18%)	9 (13%)	10 (15%)
Diabetes mellitus	61 (12%)	10 (6%)	12 (20%)	12 (20%)
Solid cancer	50 (10%)	7 (14%)	5 (10%)	5 (10%)
Hematologic disease^b^	48 (10%)	9 (19%)	4 (8%)	5 (10%)
Immunosuppression^c^	55 (11%)	11 (20%)	8 (14%)	11 (20%)

^a^Other reported co-morbidities were in descending order; renal disease (*n*=23), autoimmune disease (*n*=19), liver disease (*n*=17), splenectomy (*n*=4) and HIV (*n*=3).

^b^Hematologic disease includes hematological malignancies.

^c^Pharmacological immunosuppression was noted if the patient was treated with biological drugs, methotrexate or corticosteroids equivalent to ≥ 10 mg prednisolone.

Fifteen% of the patients (*n*=79) were treated at ICU and 33% of these individuals (*n*=26) died within 28 days. We did not see any significant difference in sepsis severity and admission to ICU between the groups divided according to invasive potential (Table 2). There was, however, a significant difference (*p*=0.015) of IPD incidence with unknown infection focus between the three groups of pneumococcal serotypes with different invasive potential. Infections with unknown foci were noted in 11.2% of cases in the group with low invasive capacity compared to 4.2% and 3.5% in the groups with intermediate and high invasive potential, respectively. Serotype 3 caused significantly more often septic shock (OR 6.33 [CI 1.59-25.29]) compared to serotype 14. Despite significance levels were not reached, septic shock was more often seen when patients were infected with serotype 19F (OR 4.38 (0.79-24.24). Furthermore, serotypes 4, 7F, 8, 9V and 18C also had an OR >2 (Table 1). Patients with IPD due to serotype 19F required significantly more often intensive care (OR 6.50 [CI 1.90-22.25]) than serotype 14. In summary, serotypes were related to incidence of septic shock and clinical outcome. Serotype 3 stood out as the most virulent serotype, and was closely followed by serotype 19F.

### Patients infected with serotypes related to a high invasive potential are younger and have fewer co-morbidities

Co-morbidities were found in 66% of the patients and cardiovascular and lung diseases dominated and were found in 27 and 24% of the individuals, respectively. Patients infected with highly invasive serotypes were younger, that is, median age was 62 years (range 19–95 years), and had a relatively low prevalence of co-morbidities (48%). The median age in the group with intermediate invasive potential was 68 years (range 20–97 years) and 61% were diagnosed with co-morbidities. The median age was 72 years (range 23–101 years) in the group with low invasive serotypes and 79% were diagnosed with co-morbidities. The differences between the groups were significant for age, co-morbidity, or two or more co-morbidities (Table 2).

Patients infected with pneumococcal serotypes 3, 6A, 6B, 18C, 19F and 23 F had significantly more co-morbidities per individual compared to patients infected with serotype 14. Patients presenting with serotypes 1 and 7F were significantly younger than individuals suffering from IPD with serotype 14 (Table 1). In conclusion, we found that highly invasive serotypes [1,5,7] caused IPD in younger and previously healthier individuals, whereas low invasive serotypes (3, 6, 8, 15, 19, 23 and 33) caused IPD merely in the elderly with more co-morbidities.

## Discussion

The virulence of different pneumococcal serotypes most probably differs significantly. Case fatality rate (CFR) is usually studied as a clinical outcome, but is biased since pneumococci with serotypes related to a low CFR infect relatively healthier and younger individuals and vice versa. Our hypothesis was that septic shock as a primary clinical outcome would give a more valid picture of the virulence since shock is a result of the immune response induced by the virulent microorganism in question.

Due to the retrospective nature of this study incomplete data sets were occasionally found in the medical records of patients with less severe disease. This may underestimate the level of severe sepsis but not likely the studied endpoint diagnosis septic shock. The groups of serotypes with high, intermediate and low invasive potential according to Brueggeman *et al*. are based on the relation of carriage and IPD in children [3]. Whether this is true also for adults have not been fully confirmed, but the classification has, however, been used in studies on adults [4,5]. In the present study, the number of isolates for each serotype was relatively low, *i.e.*, 23 serotypes represented 10.1% of the isolates. Statistical evaluation of serotypes with lower frequency was often not possible and a lack of significance for many of the serotypes may fall within a statistical type II error. Clustering of serotypes was thus necessary. Serotype 14 was chosen as a reference since it was a common serotype, and has also been proven to be temporally and geographically stable [3].

We found that patients infected with high invasive serotype pneumococci were significantly younger with fewer co-morbidities. In contrast, patients infected with low invasive potential serotypes were significantly older and had more co-morbidities. Patients infected with high invasive serotypes had lower 28-day mortality, albeit not significant. Our results thus support the theory that pneumococci related to serotypes with intermediate and low invasive potential act as opportunistic bacteria, primarily causing IPD in patient’s immunosupressed by disease or high age, whereas high invasive serogroups primarily act as primary pathogens and more frequently affect younger and healthier individuals [5,11]. In line with the findings of Jansen *et al.*, we also found that patients infected with serotypes with low invasive potential more often presented with unknown foci as a marker for more severe disease [4].

The distribution of serogroups differs from other studies, also earlier Swedish studies done in the metropolitan areas of Gothenburg and Stockholm [5,7]. These findings reflect the tendency of serogroups to vary geographically and over time [22] that emphasizes the importance of local surveillance. This issue is even more important after the introduction of pneumococcal conjugate vaccines since replacement and a shift in circulating serotypes may be expected.

Serotypes differ in their capacity to cause severe disease and IPD in various types of patients. In analogy with Garcia-Vidal *et al.*[12], we found that serotype 3 gave significantly more often septic shock and higher mortality compared to serotype 14. Serotype 3 stands out amongst most other pneumococcal serotypes since it has a large and mucoid polysaccharide capsule [11] inhibiting phagocytosis [23]. Moreover, it is particularly interesting since a hyporesponsiveness to serotype 3 after a PCV booster dose has been observed as well as a lack of protection against clinical infection after vaccination [24]. In a recent study, Dagan *et al.* did not show any decrease in nasopharyngeal carriage of serotype 3 after vaccination with PCV13 [25]. To our knowledge, no clinical data is available supporting that PCV13, where serotype 3 is included, would be protective against this particular serotype. Serotype 3 pneumococci are common colonizers and constituted five percent of the 1,128 pneumococcal nasopharyngeal disease carriage strains in Skåne 2008 (Ahl *et al*., unpublished data).

Pneumococci with serotype 19F caused more often septic shock than serotype 14, albeit not significantly, but was associated with a significantly higher percentage of patients that were admitted to the ICU (OR 6.50 [CI 1.90-22.25]) compared to serotype 14. This is in accordance with the knowledge that serotype 19F is one of the most encapsulated serotypes and is associated with a high risk of mortality [11].

We found no significant difference in frequency of septic shock and mortality between patients infected with pneumococci having serotypes with high, intermediate or low invasive potential. Since clinical sepsis is a response to infection by the host immune system, an explanation would be that patients infected with low invasive serotypes more often are immunosenescent and therefore do not clinically present severe disease. The classification of serotypes according to their invasive potential seems suboptimal for comparison of capacity to cause septic shock since serotypes 3 and 6A/6B are both classified as low invasive serotypes by Brueggeman *et al.*, and thus caused more and less septic shock, respectively, compared to serotype 14.

Future research to improve the vaccine protection for serotype 3 is probably needed and special concerns with serotype 3 have to be considered in further studies of vaccination types and policies. Larger prospective clinical and laboratory studies are required to study serotypes one by one, and to determine whether some serotypes are more virulent and cause more severe clinical disease than others. Clinical studies and/ or meta-analysis have to be large enough to be able to compensate for confounding factors such as age and co-morbidities, and to include enough numbers of uncommon serotypes. Replacement with non-vaccine pneumococcal serotypes as a consequence of vaccination is a fact [26], and therefore it is important to study all aspects of emerging serotypes.

## Conclusions

Patients infected with *S. pneumoniae* serotype 3 significantly more often suffered from septic shock compared to patients with serotype 14. Our results support the hypothesis that serotypes with high invasiveness mainly cause IPD in younger patients with less co-morbidities. In contrast, serotypes with low and intermediate invasive potential mostly cause IPD in the elderly with defined co-morbidities, and thus can be considered as opportunistic.

## Competing interest

JA is a member of Pfizer pneumococcal advisory board in Sweden.

## Authors’ contribution

JA carried out collection of data, made all the analyses, interpreted the data and prepared the manuscript as the lead writer. NL carried out collection of data, interpreted the data, drafted the manuscript and revised the manuscript. AF participated in the study design. IO and FR participated in the study design and have revised the manuscript. KR participated in the study design, drafted the manuscript and revised the manuscript. All authors read and approved the final manuscript.

## Pre-publication history

The pre-publication history for this paper can be accessed here:

http://www.biomedcentral.com/1471-2334/13/492/prepub
